# Olig2 SUMOylation protects against genotoxic damage response by antagonizing p53 gene targeting

**DOI:** 10.1038/s41418-020-0569-1

**Published:** 2020-06-01

**Authors:** Huiqing Liu, Weiji Weng, Rongjun Guo, Jie Zhou, Jun Xue, Shan Zhong, Jinke Cheng, Michael X. Zhu, Si-Jian Pan, Yong Li

**Affiliations:** 1 Department of Biochemistry and Molecular Cell Biology, Shanghai Key Laboratory for Tumor Microenvironment and Inflammation, Shanghai Jiao Tong University School of Medicine, Shanghai, 200025 China; 2 Department of Physiology, School of Basic Medicine, Kunming Medical University, Kunming, 650000 Yunnan China; 3 Department of Neurosurgery, Ruijin Hospital, Shanghai Jiao Tong University School of Medicine, Shanghai, 200025 China; 4 Department of Integrative Biology and Pharmacology, McGovern Medical School, The University of Texas Health Science Center at Houston, Houston, TX 77030 USA

**Keywords:** Transcription factors, CNS cancer

## Abstract

Posttranslational modifications of nuclear proteins, including transcription factors, nuclear receptors, and their coregulators, have attracted much attention in cancer research. Although phosphorylation of oligodendrocyte transcription factor 2 (Olig2) may contribute to the notorious resistance of gliomas to radiation and genotoxic drugs, the precise mechanisms remain elusive. We show here that in addition to phosphorylation, Olig2 is also conjugated by small ubiquitin-like modifier-1 (SUMO1) at three lysine residues K27, K76, and K112. SUMOylation is required for Olig2 to suppress p53-mediated cell cycle arrest and apoptosis induced by genotoxic damage, and to enhance resistance to temozolomide (TMZ) in glioma. Both SUMOylation and triple serine motif (TSM) phosphorylation of Olig2 are required for the antiapoptotic function. Olig2 SUMOylation enhances its genetic targeting ability, which in turn occludes p53 recruitment to *Cdkn1a* promoter for DNA-damage responses. Our work uncovers a SUMOylation-dependent regulatory mechanism of Olig2 in regulating cancer survival.

## Introduction

Glioblastoma (GBM) is the most common primary malignant brain tumor in adults, which displays notorious resistance to conventional therapy [1]. Transcription factors (TFs) have been shown to be critical in tumorigenesis and therapeutic resistance in GBM cells, which collectively contribute to the initiation of tumors [2–4]. In addition, the oligodendrocyte TF 2 (Olig2) has been reported to be critical in promoting proliferation, migration/invasion, and resistance to radio-/chemotherapy of glioma cells [5–7].

Olig2 is a basic helix–loop–helix (bHLH) TF expressed in neural progenitor cells (NPCs) and oligodendrocyte progenitor cells during development of the central nervous system (CNS) [8]. Olig2 plays two contrasting roles during CNS development: early on, it sustains the replication competence of NPCs, and later, it promotes cell cycle exit and specifies the formation of oligodendrocyte lineage cells from proliferating NPCs [5, 9, 10].

Olig2 is phosphorylated at S10, S13, and S14 (also known as the triple serine motif, TSM) [11]. The phosphorylation at these sites is developmentally regulated and controls the proliferative functions of Olig2 in NPCs [11, 12]. In addition, TSM-phosphorylated Olig2 promotes tumor growth in a genetically defined murine glioma model [11]. This tumorigenic function of Olig2 is correlated with its oppositional relationship to p53, where wild-type (WT) Olig2 and the triple phospho-mimetic (TPM) Olig2 (S10D/S13E/S14E) repress the irradiation-induced p53 activation and expression of *Cdkn1a* (also known as *p21*) [6]. Moreover, a recent study showed that TSM phosphorylation serves as a regulator that switches glioma from proliferation to invasion status [7].

Posttranslational modifications (PTMs) of nuclear proteins, including TFs, nuclear receptors, and their coregulators, represent a mechanism frequently used by the cell to respond to environmental changes [13, 14]. It would be of significance to identify additional PTMs on Olig2 and learn how they interact with phosphorylation to regulate Olig2 functions. Here, we show that Olig2 is SUMOylated, a form of PTMs involving covalent enzymatic conjugation of small ubiquitin-like modifier (SUMO) proteins to specific lysine residues of substrate proteins [14]. We unveiled the critical involvement of Olig2 SUMOylation in overcoming cell cycle arrest and apoptosis in response to genotoxic drugs. Olig2 SUMOylation protects cells against genotoxic damage by disrupting the recruitment of p53 to *Cdkn1a* promoter. Thus, SUMOylation is important for Olig2 to function as an antiapoptotic factor in genotoxic stress.

## Materials and methods

### Antibodies and reagents

The following primary antibodies and reagents were used: mouse anti-Olig2 (MABN50, Millipore), mouse anti-SUMO1 (33-2400, Thermo Fisher Scientific), mouse anti-BrdU (sc-32323, Santa Cruz), mouse anti-p53 (sc-126, Santa Cruz), rabbit anti-p53 (ab32389, Abcam), mouse anti-Flag (F1804, Sigma), mouse anti-Myc (sc-40, Santa Cruz), rabbit anti-Olig2 (AB9610, Millipore), rabbit anti-SUMO1 (4940, Cell Signaling Technology), rabbit anti-cleaved caspase-3 (9661, Cell Signaling Technology), rabbit anti-gamma H2AX (phospho S139) (ab2893, Abcam), rabbit anti-Ki67 (ab16667, Abcam), mouse anti-phosphoserine (ab6639, Abcam), rabbit anti-Olig2 (phospho S10 + S13 + S14) (ab183487, Abcam), rabbit anti-Histone H3 (4499, Cell Signaling Technology), rabbit anti-acetyl-p53 (Lys379) (2570, Cell Signaling Technology), rabbit anti-p21 (ab218388, Abcam), rabbit anti-HA (H6908, Sigma), and SUMOylation 1 Affinity Beads (ASM11, Cytoskeleton). Etoposide (ETO, E1383), temozolomide (TMZ, T2577), BrdU (B5002), and tamoxifen (TAM, T5648) were purchased from Sigma. CHIR-99021 (S2924) was purchased from Selleck.

### Cells and human GBM specimen

HEK 293T, Neuro-2a, U87-MG, and HCT-116 cells were cultured in Dulbecco’s Modified Eagle’s Medium (DMEM) supplemented with 10% fetal bovine serum and maintained at 37 °C in a 5% CO_2_ humidified incubator. Cells were transfected at 80–90% confluency using Lipofectamine 3000 (Invitrogen) according to the manufacturer’s instructions.

Human GBM tissue was acquired from Ruijin Hospital (Shanghai, China) with the approval by Ethics Committee for Clinical Trial and Medical Devices of Ruijin Hospital. Informed consent was obtained from all subjects.

### Plasmids

His-SUMO1 and HA-CBP plasmids were gifts from Jianxiu Yu and Zhaoyuan Hou (Shanghai Jiao Tong University School of Medicine, Shanghai, China), respectively [15, 16]. Mouse Olig2, Hdac1, p53, and Senp2 cDNA were amplified by PCR from mouse brain tissue and inserted into p3×Flag-Myc-CMV24 (Sigma), pCDNA3.1/Myc-His(−) (Invitrogen), pEGFP-C1 (Clontech), and pCMV-HA (Clontech) vectors to obtain Flag-Olig2, Myc-Olig2, Myc-Hdac1, Flag-p53, GFP-Senp2, and HA-Senp2, respectively. HA-Sox10, HA-Olig1, and HA-Nkx2.2 were generated by inserting mouse Sox10, Olig1, and Nkx2.2 cDNA into pCDNA3 vector with an HA tag at the C-terminus, respectively. Myc-Sirt1 was generated by inserting human Sirt1 cDNA into pCDNA3.1/Myc-His(−) vector. Mutations of Flag/Myc-Olig2, His-SUMO1, and GFP-Senp2 were generated using PCR-directed mutagenesis and all mutations were confirmed by DNA sequencing. For luciferase reporter assay, human *Cdkn1a* promoter fragment and mouse *Mycn*-promoter fragment [17, 18] were amplified and cloned into pGL3-Basic vector (Promega). For lentivirus packaging, mouse Olig2 WT or 3KR cDNA was inserted into pCDH-CMV-MCS-EF1-copGFP vector (System Biosciences), with fused Myc and His tags at the C-terminus.

### Immunoprecipitation

Co-immunoprecipitation (Co-IP) under nondenaturing condition was performed as previously described [19]. Briefly, cells were lysed with whole cell lysis buffer (20 mM Tris-HCl, pH 7.5, 150 mM NaCl, 1% Triton X-100, 0.1% SDS, 2 mM EDTA, 10% glycerol) supplemented with protease inhibitor cocktail (Sigma), phosphatase inhibitor cocktail (Selleck), and 20 mM N-ethylmaleimide (Sigma). After centrifugation for 15 min at 13,000 × *g*, supernatant was collected and the indicated antibody was added. Immunoprecipitants were collected with protein A/G agarose (Pierce), washed three times, boiled in 2× SDS loading buffer (50 mM Tris-HCl pH 6.8, 4% 2-mercaptoethanol, 20% glycerol, 4% SDS), and subjected to western blotting analysis.

To detect SUMOylation, immunoprecipitation under denaturing conditions (De-IP) was performed as described previously [20]. Cells or tissues (mouse spinal cord or human GBM) were lysed and homogenized in SDS lysis buffer (50 mM Tris-HCl, pH 6.8, 40 mM dithiothreitol, 5% glycerol, 2% SDS) supplemented with protease inhibitor cocktail and 20 mM N-ethylmaleimide. Lysates were then boiled at 95 °C for 15 min and diluted to the final concentration of 0.2% SDS with dilution buffer (50 mM Tris-HCl, pH 7.5, 150 mM NaCl, 1% Nonidet P-40) supplemented with protease inhibitor cocktail and 20 mM N-ethylmaleimide. Debris was removed by centrifugation and the indicated antibody was added. After washing, immunoprecipitants were boiled in 2× SDS loading buffer and subjected to western blotting analysis with indicated antibodies.

### Identification of Olig2 SUMOylation site by mass spectrometry

HEK 293T cells transfected with Flag-Olig2 and His-SUMO1 T95R were lysed with whole cell lysis buffer (20 mM Tris-HCl, pH 7.5, 150 mM NaCl, 1% Triton X-100, 0.1% SDS, 2 mM EDTA, 10% glycerol) supplemented with protease inhibitor cocktail, phosphatase inhibitor cocktail, and 20 mM N-ethylmaleimide. After incubation with the anti-Flag antibody at 4 °C overnight, protein A/G beads were added. After 1-h incubation at 4 °C, immunoprecipitants were washed sequentially with whole cell lysis buffer for four times, and with 5 mM NH_4_HCO_3_ for two times at 4 °C. Proteins were eluted from the beads with 0.15% trifluoroacetic acid (Sigma). After centrifugation at 4 °C for 15 min at 13,000 × *g*, the supernatant was collected and dried in vacuum. Eluted proteins were redissolved in 25 mM NH_4_HCO_3_ followed by digestion with trypsin (Promega) at 37 °C overnight. The supernatant was dried before LC–MS/MS analysis.

The samples were resuspended with 20 mL Buffer A (water with 0.1% formic acid) and analyzed by on-line nanospray LC–MS/MS on a Q Exactive HF (Thermo Fisher Scientific, Waltham, MA, USA) coupled to an Acquity UPLC M-class (Waters Corporation, Milford, MA, USA). Three microliters of peptide was loaded (analytical column: Waters nanoEase M/Z HSS C18 T3, 75 μm × 25 cm) and separated with a 60 min linear gradient, from 4 to 30% Buffer B (acetonitrile with 0.1% formic acid). The column flow rate was maintained at 500 nL/min with the column temperature of 40 °C. The electrospray voltage of 2 kV versus the inlet of the mass spectrometer was used.

The mass spectrometer was run under data dependent acquisition mode, and automatically switched between MS and MS/MS mode. The parameters were: (1) MS: scan range (*m*/*z*) = 350–1600; resolution = 60,000; AGC target = 3e6; maximum injection time = 50 ms; include charge states = 2–7; (2) HCD-MS/MS: resolution = 15,000; isolation window = 1.6; AGC target = 1e5; maximum injection time = 100 ms; collision energy = 30.

Tandem mass spectra were processed by PEAKS Studio version X (Bioinformatics Solutions Inc, Waterloo, ON, Canada). PEAKS DB was set up to search the uniprot_proteome_mus_musculus_201907 database (ver 201907, 22290 entries) assuming trypsin as the digestion enzyme. PEAKS DB was searched with a fragment ion mass tolerance of 0.02 Da and a parent ion tolerance of 7 ppm. Oxidation (M), carbamidomethylation (C), N-ethylmaleimide (C), nethylmaleimidehydrolysis (C), and GG modification (K) were specified as the variable modifications. The peptides with −10lgP ≥ 20 and the proteins with −10lgP ≥ 20 and containing at least one unique peptide were filtered.

### Cycloheximide (CHX) chase assays

Protein degradation was assessed by CHX (Sigma) chase assay as described previously [19]. CHX was added to the culture medium (20 μg/mL final concentration) 24 h post transfection, samples were taken at the indicated time points, and steady-state levels of protein of interest were determined by western blotting with appropriate antibodies as indicated.

### Nuclear extraction

Nuclear fraction was isolated as previously described with minor modifications [12]. Briefly, cell pellets were resuspended with hypotonic buffer (20 mM Tris-HCl, pH 7.4, 10 mM NaCl, 3 mM MgCl_2_). Homogenates were incubated on ice for 15 min and then 10% Nonidet P-40 was added to a final concentration of 0.5%. After centrifugation at 700 × *g* for 10 min, supernatant was collected as the cytoplasmic fraction. The pellet was washed with hypotonic buffer and then lysed with whole cell lysis buffer (20 mM Tris-HCl, pH 7.5, 150 mM NaCl, 1% Triton X-100, 0.1% SDS, 2 mM EDTA, 10% glycerol). After centrifugation at 13,000 × *g* for 10 min, supernatant was taken as the nucleus fraction. Protein concentration was quantitated using a BCA assay kit (Thermo Fisher Scientific). All buffers were supplemented with protease inhibitor cocktail, phosphatase inhibitor cocktail, 1 mM PMSF, and 0.5 mM dithiothreitol.

### Luciferase reporter assay

Construction of the plasmids for luciferase reporter assay was described above. The *Cdkn1a*- or *Mycn*-promoter luciferase plasmids were cotransfected with *Renilla* luciferase plasmid, WT Flag-Olig2, 3KR Flag-Olig2, AQ Flag-Olig2, and/or His-SUMO1 into Neuro-2a cells using Lipofectamine 3000. At 24 h post transfection, cells were harvested and analyzed for the luciferase activity using a dual-luciferase reporter assay kit (Promega) following the manufacturer’s instructions. The luciferase activity for each group was normalized with *Renilla* luciferase activity. See also [5].

### Lentivirus packaging

Olig2-expressing lentiviral constructs were generated as described above. Lentivirus packaging was performed by OBiO Technology Co. Ltd (Shanghai, China). U87-MG cells were stably infected with Lenti-GFP (1.63 × 10^9^ TU/mL), Lenti-Olig2^WT^ (1.38 × 10^9^ TU/mL), and Lenti-Olig2^3KR^ (1.17 × 10^9^ TU/mL), respectively, [21].

### TUNEL staining

TUNEL staining was performed using an *in situ* cell death detection kit (Roche) following the manufacturer’s instructions. Briefly, cells were fixed with 4% paraformaldehyde/4% sucrose and permeabilized in 0.1% Triton X-100 in phosphate-buffered saline (PBS). After washing with PBS, cells were then incubated in the reaction solution containing terminal deoxynucleotidyl transferase and nucleotide mixture at 37 °C for 1 h. See also [22].

### Immunofluorescence and confocal microscopy

Immunofluorescence staining was performed as described previously [20]. Briefly, cells were fixed with 4% paraformaldehyde/4% sucrose, blocked in 5% goat serum and 0.1% Triton X-100 (in PBS), and incubated with indicated primary antibodies overnight. Cells were then rinsed in PBS three times and incubated with appropriate fluorescence-conjugated secondary antibodies. After washing with PBS three times, cells were incubated with DAPI and mounted using Fluoromount-G (SouthernBiotech). For BrdU staining, cells were incubated with 10 μM BrdU for 1 h prior to fixation. After fixation, cells were incubated in 2 M HCl for 10 min at room temperature, followed by neutralization with PBS. Confocal images were obtained using a Leica SP8 confocal microscopy system (Leica Microsystems Inc., Buffalo Grove, IL, USA).

### Chromatin immunoprecipitation (ChIP)

ChIP was performed using an EZ-ChIP chromatin immunoprecipitation kit (Millipore) with minor modifications [21]. In brief, ETO-treated cells were fixed with 1/10 volume of 11% formaldehyde solution (50 mM HEPES-KOH, pH 7.5, 100 mM NaCl, 1 mM EDTA, 0.5 mM EGTA, 11% formaldehyde). Cells were then harvested and lysed in SDS lysis buffer (50 mM Tris-HCl, pH 8.1, 10 mM EDTA, 1% SDS) supplemented with protease inhibitor cocktail and 20 mM N-ethylmaleimide. The lysates were sonicated and centrifugated to remove cellular debris. Samples were diluted with ChIP dilution buffer (16.7 mM Tris-HCl, pH 8.1, 167 mM NaCl, 1.2 mM EDTA, 0.01% SDS, 1.1% Triton X-100) supplemented with protease inhibitor cocktail and 20 mM N-ethylmaleimide, after which 1% input was taken. DNA/protein complex was immunoprecipitated with the anti-Flag antibody or anti-p53 antibody. Normal mouse IgG was used as the negative control. Immunoprecipitants were pulled down with ChIP blocked protein G agarose (Millipore) and then washed sequentially with low salt wash buffer (20 mM Tris-HCl, pH 8.1, 150 mM NaCl, 2 mM EDTA, 0.1% SDS, 1% Triton X-100), high salt wash buffer (20 mM Tris-HCl, pH 8.1, 500 mM NaCl, 2 mM EDTA, 0.1% SDS, 1% Triton X-100), LiCl wash buffer (10 mM Tris-HCl, pH 8.1, 0.25 M LiCl, 1 mM EDTA, 1% Nonidet P-40, 1% deoxycholic acid), and TE buffer (10 mM Tris-HCl, pH 8.0, 1 mM EDTA). Samples were then eluted with elution buffer (1% SDS, 100 mM NaHCO_3_). Eluates were incubated at 65 °C overnight in the presence of 200 mM NaCl to reverse the crosslinked DNA. Free DNA was purified with a DNA purification kit (Tiangen, Beijing, China), followed by quantitative PCR in LightCycler 480 PCR system (Roche, Basel, Switzerland) using SYBR Green mix (Vazyme, Nanjing, China).

Primers used for the ChIP assay were as follows: human *Cdkn1a* distal forward GGGCTTTCCACCTTTCACC, reverse ACCATCCCCTTCCTCACCT; proximal forward GGGCAGGAGGCAAAAGTCCT, reverse GAAGCCTGTCCTCCCCGAGG; non-target forward TCTGTGAAAACATGCCCAGC, reverse TTGAAACAGGGGACCGTGTC; human *Bax* forward CTCTCGGACCCTCGAGAAC, reverse AGGCTGGGCCTGTATCCTAC; mouse *Cdkn1a* forward AGGTCAGCTAAATCCGAGGAGGAA, reverse TCCTGCTTTGGAGAAGCTGTAGT; *Egfr* forward GTGGTCCCAATTTCCTGCTG, reverse ATGGAGCTCATGGACCTCATTG; *Fgfr3* forward TAGACCCCCACCGAAGTCAA, reverse, GACTGTCTACCAGCACGCTT; *Tgfb2* forward, CTCCTGCAGCTCTGTTGTGA, reverse TTTATTTCTTTGCTTGCTTGCTTT.

### Quantitative PCR

Total RNA was extracted from ETO-treated Neuro-2a cells using TRNzol-A+ reagent (Tiangen, Beijing, China) as described previously [23]. An aliquot of RNA (2 µg) was used for cDNA synthesis using PrimeScript RT reagent kits with gDNA Eraser (Takara). cDNA was then analyzed in LightCycler 480 PCR system (Roche, Basel, Switzerland) using SYBR Green mix (Vazyme, Nanjing, China). The primer for *Cdkn1a* quantitation was as follows: mouse *Cdkn1a* forward CCTGGTGATGTCCGACCTG, reverse CCATGAGCGCATCGCAATC; human *Cdkn1a* forward TGTCCGTCAGAACCCATGC, reverse AAAGTCGAAGTTCCATCGCTC.

### Colony formation assay

U87-MG cells stably expressing GFP vector, WT Myc-Olig2, or 3KR Myc-Olig2 were plated at 1000 cells per 60 mm dish, followed by treatment with 200 μM TMZ for 24 h, after which cells were allowed to grow for 6–7 days in normal culture medium. Colonies were fixed with 4% paraformaldehyde and stained with 0.1% crystal violet for 15 min. One colony is defined to consist of over 50 cells [24].

### Animal experiments

A total of 2 × 10^6^ U87-MG cells were injected into the flank of 6-week-old BALB/c nude mice to generate model of GBM [25]. When tumors reached the size of approximal 500 mm^3^, animals were treated with TMZ (40 mg/kg, i.p) for 5 consecutive days and tumor size was monitored every 3 days. Tumor volumes were calculated using the formula (length × width^2^)/2.

To generate intracranial GBM xenografts, 5 × 10^5^ U87-MG cells in DMEM (5 μL) were injected into the brains of 6-week-old BALB/c nude mice at a rate of 0.2 μL/min. The coordinates are as follows: 2 mm lateral (right), 1 mm anterior, and 3 mm ventral (according to bregma). Animals were treated with TMZ (40 mg/kg, i.p) for 5 consecutive days after 15 days post transplantation.

For histological analysis and immunostaining of orthotopically grafted tumors, mice were sacrificed 5–7 days post TMZ administration. Brains were fixed with 4% paraformaldehyde, cryoprotected in 30% sucrose, and embedded in OCT (TissueTek, Sakura). Cryostat sections (12 μm thickness) were made and used for staining.

All animal protocols were in accordance with the guidelines established by the Care and Use of Laboratory Animals of Shanghai Jiao Tong University School of Medicine and approved by the Institutional Animal Care and Use Committee.

### Statistical analysis

All data are presented as means ± s.e.m. of at least three independent experiments, with statistical significance assessed by Student’s *t* test for two group comparison, one-way ANOVA or two-way ANOVA with Tukey’s multiple comparison test for multiple group comparisons. Statistical significance is defined as *p* < 0.05. (*p < 0.05, **p < 0.01, ***p < 0.001.)

## Results

### Olig2 is modified by SUMO1

**Fig. 1:**
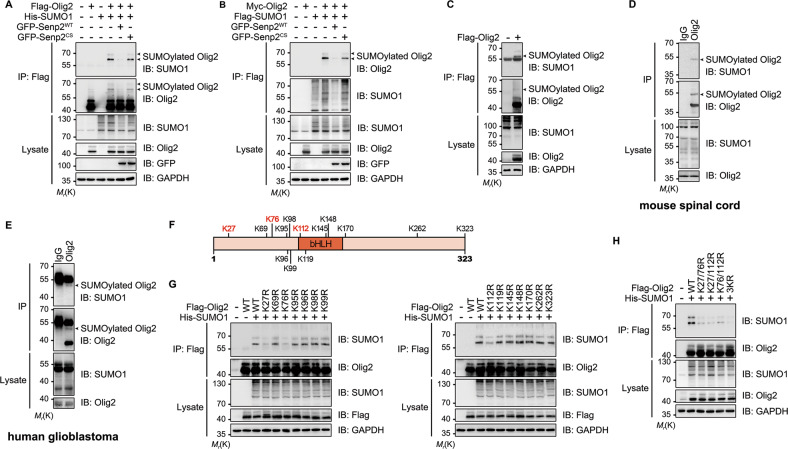
Olig2 is modified by SUMO1 at K27, K76, and K112. **a** SUMO1 modification of Olig2 in HEK 293T cells. HEK 293T cells were transfected with Flag-Olig2, His-SUMO1, GFP-Senp2^WT^, or GFP-Senp2^CS^. Cell lysates were prepared under denaturing conditions and immunoprecipitated with anti-Flag antibody and then immunoblotted using rabbit anti-Olig2 and rabbit anti-SUMO1 antibodies. **b** Similar to **a** for reciprocal immunoprecipitation. HEK 293T cells were transfected with Myc-Olig2, Flag-SUMO1, GFP-Senp2^WT^, or GFP-Senp2^CS^. Cell lysates were immunoprecipitated using anti-Flag antibody and then immunoblotted with rabbit anti-Olig2 and rabbit anti-SUMO1 antibodies. **c** Olig2 is modified by endogenous SUMO1. HEK 293T cells were transfected with or without Flag-Olig2. Cell lysates were immunoprecipitated with anti-Flag antibody, followed by immunoblotting using rabbit anti-Olig2 and rabbit anti-SUMO1 antibodies. **d**, **e** Olig2 is modified by SUMO1 in vivo. Mouse spinal cord tissue or human glioblastoma tissue were homogenized and lysed under denaturing conditions, followed by immunoprecipitation with mouse anti-Olig2 antibody. Rabbit anti-SUMO1 and rabbit anti-Olig2 antibodies (**d**) or mouse anti-SUMO1 and mouse anti-Olig2 antibodies (**e**) were used for western blotting analysis. **f** Schematic showing lysine residues in mouse Olig2. **g**, **h** Identification of Olig2 SUMOylation sites. The indicated K → R mutants were transfected into HEK 293T cells with His-SUMO1. Cell lysates were subsequently immunoprecipitated using anti-Flag antibody and immunoblotted with rabbit anti-Olig2 and rabbit anti-SUMO1 antibodies. For all panels, arrowheads indicate SUMOylated Olig2. Blot images shown are representative of at least three independent experiments.

Many TFs or coregulators of transcription are SUMO substrates, and in most cases, modification with SUMO results in transcriptional repression [26, 27]. De-IP analysis showed that the anti-Flag antibody pulled down, in addition to Flag-Olig2 (~42 kD), two higher molecular weight bands ~18 and 25 kD larger than the unmodified Olig2 protein, indicating that Olig2 is SUMOylated. Reciprocally, the anti-Flag antibody also pulled down the similar two higher molecular weight bands detectable by the anti-Olig2 antibody (Fig. 1b). Importantly, the SUMOylated Olig2 bands were abolished by co-expression of the SUMO deconjugating enzyme GFP-Senp2 (Senp2^WT^), but not the catalytically dead Senp2 mutant (Senp2^CS^, C547S) (Fig. 1a, b).

Next, to test whether Olig2 is conjugated by endogenous SUMO1, lysates from HEK 293T cells transiently transfected with Flag-Olig2 were subjected to De-IP and immunoblotted with the anti-SUMO1 and anti-Olig2 antibodies. In both cases, a band corresponding to the predicted size of SUMO1-conjugted Olig2 was detected (Fig. 1c). Using De-IP in lysates prepared from mouse spinal cord tissue and human GBM, we detected a weak but nonetheless clear band of ~52 kD, which corresponds to the estimated molecular weight of SUMOylated Olig2 (~37 kD for the endogenous Olig2 plus ~15 kD for SUMO1) (Fig. 1d, e). In SUMOylated proteins enriched by SUMO1 affinity beads from mouse spinal cord lysates, we also observed the same sized band (Fig. S1A). With the addition of the Flag epitope, Flag-Olig2 becomes ~5 kD larger (net weight of ~42 kD) than endogenous Olig2 (Fig. S1B), giving rise to the SUMOylated Flag-Olig2 of ~57 kD (Fig. 1c). Because of the additional amino acids added in the epitope-tagged SUMO1, the SUMOylated-Olig2 appeared even larger (~60 kD) in Fig. 1a, b. The weaker upper band detected in Fig. 1a, b probably represents additional PTM of the SUMOylated-Olig2 species, which may be too weak to be detectable in the absence of SUMO1 overexpression (Figs. 1c–e and S1A). Taken together, these results suggest that Olig2 is SUMOylated both in vitro and in vivo.

### K27/K76/K112 are the major SUMOylation sites in Olig2

Protein SUMOylation typically occurs at lysine residues located within the consensus sequence Ψ-K-X-E, where Ψ is any hydrophobic amino acid and X is any amino acid [28]. However, no consensus SUMOylation sequence was found in Olig2 with the use of three SUMOylation prediction programs, GPS-SUMO [29], SUMO plot (http://www.abgent.com/sumoplot), and JASSA (Fig. S1C) [30]. Given that there are only 14 lysine residues in mouse Olig2 (Fig. 1f), we decided to substitute all of them individually with arginines and then test how the mutations affect SUMO1 conjugation. Among the 14 substitutions, only K27R, K76R, and K112R resulted in marked reduction, but not complete loss, of Olig2 SUMOylation, as compared with WT Olig2 (Fig. 1g). To confirm these modifications, we purified Olig2 proteins co-expressed with His-SUMO1 T95R by De-IP and subjected them to LC–MS/MS analysis. The T95R substitution allows SUMO1 to be cleaved by trypsin to generate a diglycine (GG) tag conjugated on the SUMOylated lysine [31, 32]. The results showed a GG tag on tryptic peptides containing K27 and K76, confirming that K27 and K76 are SUMOylated (Fig. S2A, B). To test the contribution of K112, as well as K27 and K76, to the overall SUMOylation of Olig2, we made double mutations, i.e., K27/76R, K27/112R, and K76/112R, as well as a triple mutation, K27/76/112R (3KR). All double mutations resulted in marked reduction but not complete loss of Olig2 SUMOylation, whereas 3KR exhibited the lowest level of SUMOylation among all constructs tested (Figs. 1h and S1E). Importantly, K27, K76, and K112 are well-conserved among mammalian Olig2 orthologs (Fig. S1D). Based on findings, we conclude that Olig2 is SUMOylated at K27, K76, and K112.

### Olig2 SUMOylation is required for inhibition of DNA-damage response

**Fig. 2:**
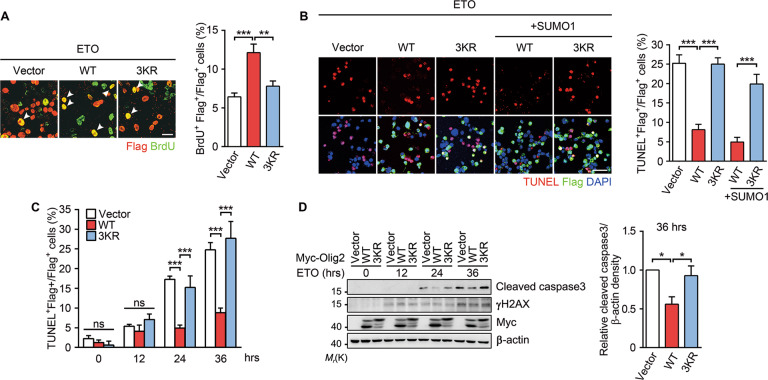
Olig2 SUMOylation is required for inhibition of DNA-damage response. **a** Representative images of BrdU^+^ (green) uptake in Flag-Olig2 expressing (red) Neuro-2a cells. Cells transfected with vector, WT, or 3KR Flag-Olig2 were incubated with 20 μM etoposide (ETO) for 36 h and then pulsed with 10 μM BrdU for 1 h. Immunostaining was performed using anti-BrdU and anti-Flag antibodies. The bar graphs represent the percentage of BrdU^+^/Flag^+^ cells. Scale bar = 25 μm. **b** Representative images of TUNEL (red) staining in Flag-Olig2 expressing (green) Neuro-2a cells. Cells transfected with vector, WT, or 3KR Flag-Olig2 were incubated with 20 μM ETO for 36 h. Cells were then subjected to TUNEL staining and immunostaining using anti-Flag antibody. The bar graphs represent the percentage of TUNEL^+^ among Flag^+^ cells. Scale bar = 50 μm. **c**, **d** WT Olig2 represses genotoxic drug-induced apoptosis. Neuro-2a cells overexpressing vector, WT, or 3KR Myc-Olig2 were incubated with 20 μM ETO for indicated periods. Cells were then subjected to TUNEL staining (**c**) or lysed for western blotting analysis using anti-cleaved caspase-3 and anti-γH2AX antibodies (**d**). Results shown are representatives of at least three independent experiments. Bar graphs are presented as means ± s.e.m. of at least three independent experiments. Statistical significance was assessed by one-way ANOVA with Tukey’s multiple comparison test (**a**, **b**, **d**) or two-way ANOVA (**c**) for multiple group comparisons. *p < 0.05; **p < 0.01; ***p < 0.001; ns nonsignificant. Note: the green fluorescence signal of cells expressing the Flag vector is always much weaker than that of cells expressing Flag-Olig2. In this series of experiments, including Figs. 2b, 2c, 5a and S3A, we routinely use the same low laser power and other parameter settings of our confocal system to acquire images for all samples.

Olig2 is reported to be a protective TF against p53-mediated cell cycle arrest and apoptosis upon irradiation-induced genotoxic damage [6]. In neuroblastoma Neuro-2a cells treated with ETO, a widely used antitumor agent [33], the number of BrdU^+^ cells was significantly higher in cells that overexpressed WT Olig2 than those transfected with the 3KR mutant (Fig. 2a), which argues for an essential role of SUMOylation in the protective effect of Olig2 against cell cycle arrest. To assess the functional significance of SUMOylation on the ability of Olig2 against tumor apoptosis, we measured apoptotic responses of the ETO-treated Neuro-2a cells. As shown in Fig. 2b, c, overexpression of WT Olig2 in Neuro-2a cells resulted in a decrease of TUNEL^+^ cells, consistent with previous studies [6, 12]. However, overexpression of 3KR failed to show any protection no matter if SUMO1 was co-expressed or not (Fig. 2b, c). On the other hand, overexpression of WT Olig2 resulted in a decrease in the cleaved caspase-3 in cells treated with ETO for 24 and 36 h, while that of 3KR failed to show any protection (Fig. 2d). These data further support the conclusion that SUMOylation is required for the protection of Olig2 against apoptosis.

### SUMOylation is required for Olig2-mediated resistance to TMZ in glioma

**Fig. 3:**
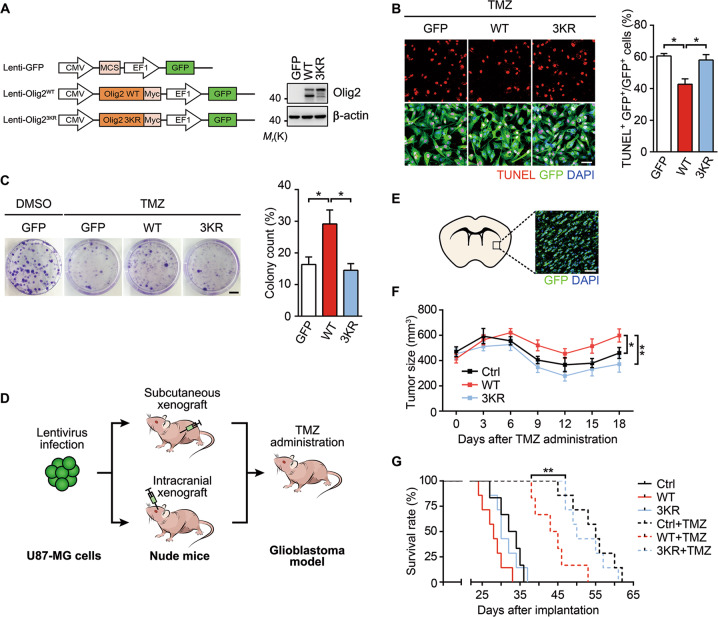
Olig2 SUMOylation enhances tumor resistance to TMZ. **a** Schematic diagram for the design of Lenti-GFP, Lenti-Olig2^WT^, and Lenti-Olig2^3KR^. Expression of WT or 3KR Myc-Olig2 in U87-MG cells was identified by western blotting. **b** Representative images of TUNEL (red) staining in Lenti-Olig2 expressing (green) U87-MG cells. Cells expressing GFP, WT, or 3KR Olig2 were incubated with 200 μM temozolomide (TMZ) for 36 h followed by TUNEL staining. The bar graphs represent the percentage of TUNEL^+^ among GFP^+^ cells. Scale bar = 50 μm. **c** Olig2 SUMOylation enhances TMZ resistance in vitro. U87-GFP, U87-Olig2^WT^, or U87-Olig2^3KR^ cells were plated at the density of 1000 cells per 60-mm dish and incubated with vehicle (DMSO) or 200 μM TMZ for 24 h, after which cells were allowed to grow for 6–7 days in normal culture medium. Colony counts are quantified after staining with 0.1% crystal violet. **d** Schematic protocol showing the generation of subcutaneous and orthotopic xenograft models of glioblastoma. **e** Representative image showing the distribution of grafted U87-MG cells (GFP^+^, green) in the mouse brain. **f** TMZ inhibits the growth of gliomas expressing GFP or 3KR Olig2, but not those expressing WT Olig2. U87-GFP, U87-Olig2^WT^, or U87-Olig2^3KR^ cells (2 × 10^6^) were subcutaneous injected into 6-week-old nude mice (Ctrl, *n* = 6; WT, *n* = 6; 3KR, *n* = 5). When tumor size reached the volume of about 500 mm^3^, mice were administered with 40 mg/kg TMZ for 5 consecutive days, and tumor size was then evaluated every 3 days. **g** Kaplan–Meier survival analysis of intracranial glioblastoma model mice. U87-GFP, U87-Olig2^WT^, or U87-Olig2^3KR^ cells (5 × 10^5^) were intracranially injected into 6-week-old nude mice (Ctrl, *n* = 6; WT, *n* = 7; 3KR, *n* = 7; Ctrl + TMZ, *n* = 7; WT + TMZ, *n* = 6; 3KR + TMZ, *n* = 7). At 15 days post transplantation, mice were administered with 40 mg/kg TMZ for 5 consecutive days. Summary graphs are presented in means ± s.e.m. Statistical significance was assessed by one-way ANOVA (**b**, **c**) or two-way ANOVA test for multiple group comparisons (**f**) or log-rank test for survival analysis (**g**). *p < 0.05; **p < 0.01.

TMZ, a DNA alkylating agent, is now a standard-of-care chemotherapeutic agent for adult patients with recurrent high-grade glioma [1]. In order to test whether Olig2 SUMOylation plays a role in TMZ resistance of glioma, we employed human GBM U87-MG cells which endogenously express WT p53 but not Olig2 (Fig. S1B) [21, 34]. U87-MG cell lines stably expressing, respectively, WT and 3KR Myc-Olig2 at comparable levels were established (Fig. 3a) and then treated with TMZ. Consistently, disruption of the three SUMOylation sites in Olig2 also abolished its protective effect against genotoxic damage (Fig. 3b). In addition, while the expression of WT Olig2 increased viability of TMZ-treated cells (Fig. 3c), the expression of 3KR did not have such an effect (Fig. 3c). To assess the function of Olig2 SUMOylation in vivo, we injected the stable U87-MG cells either subcutaneously or intracranially into nude mice as illustrated (Fig. 3d, e). The animals were treated with TMZ and then monitored for either the development of tumor or survival. In the subcutaneous xenograft model, despite beginning with similar average tumor sizes between 0 and 3 days after the TMZ treatment, the 3KR expressing gliomas showed marked decreases in size similar to controls (mice receiving cells that expressed GFP); however, the WT Olig2-expressing gliomas maintained their tumor size to a large extend (Fig. 3f). In the intracranial xenograft model, TMZ administration greatly improved the survival of mice bearing either control (GFP) or 3KR expressing tumors, with the prolonged median survival of 50 days; however, this effect was compromised by the expression of WT Olig2, resulting in a reduction of median survival to 44 days (Fig. 3g).

**Fig. 4:**
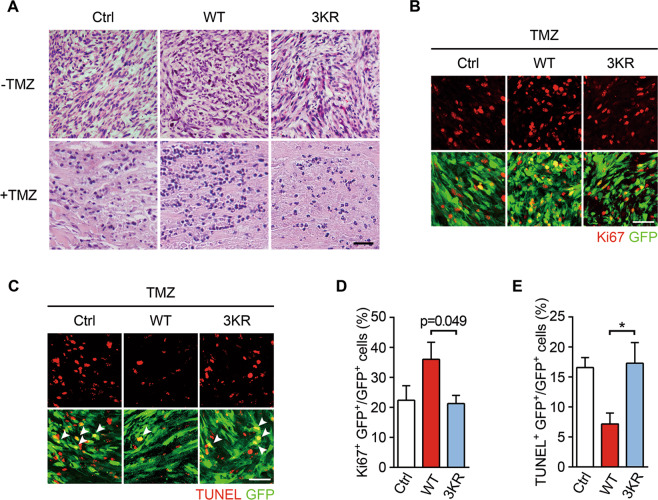
Olig2 SUMOylation inhibits apoptosis in orthotopic xenografts. Intracranial glioblastoma models of Olig2-expressing tumors were created in mice as described in Fig. 3d. Transplanted nude mice were treated with saline (−TMZ) or TMZ (+TMZ) and sacrificed 5–7 days later. **a** Representative images of H&E staining of the tumors. Frozen sections were prepared and subjected to H&E staining. Scale bar = 50 μm. **b**, **d** Olig2 SUMOylation promotes tumor proliferation after TMZ administration. Proliferating cells were labeled by Ki67 staining. The bar graph in **d** shows the percentage of Ki67^+^ (red) among GFP^+^ (green) cells. Scale bar = 50 μm. **c**, **e** Olig2 SUMOylation inhibits tumor apoptosis after TMZ administration. Cells undergoing apoptosis were labeled by TUNEL staining. The bar graph in **e** shows the percentage of TUNEL^+^ (red) among GFP^+^ (green) cells. Images shown are representatives of at least three independent experiments. Bar graphs are presented as means ± s.e.m. of at least three independent experiments. Statistical significance was assessed by one-way ANOVA with Tukey’s multiple comparison test. *p < 0.05.

In addition, H&E stains of tumor sections showed that orthotopic grafts expressing 3KR Olig2 had much more severe cell loss as compared with those expressing WT Olig2 (Fig. 4a). Tumors expressing WT Olig2 showed a higher proliferation rate (Fig. 4b, d) and a lower apoptosis rate (Fig. 4c, e) than the control counterpart and those expressing 3KR Olig2. Taken together, our results demonstrate that SUMOylation constitutes a pivotal step of Olig2 PTMs for establishing the TMZ resistance in GBM and disrupting Olig2 SUMOylation may have survival benefit in glioma therapy.

### Both SUMOylation and TSM phosphorylation are required for the antiapoptotic function of Olig2

**Fig. 5:**
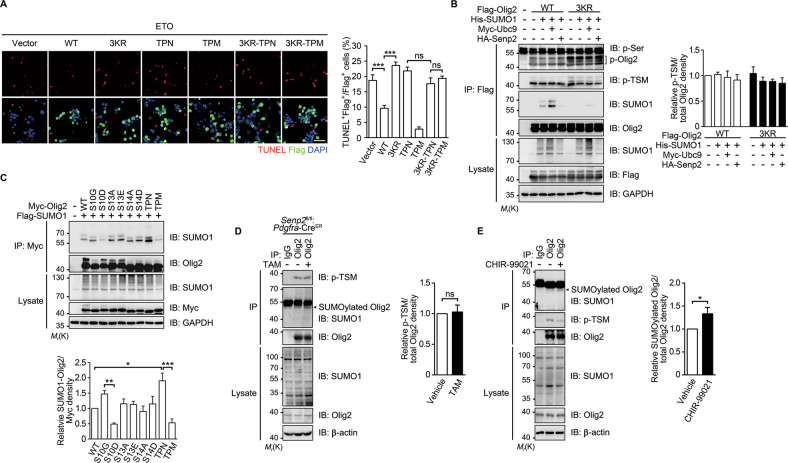
Crosstalk between Olig2 SUMOylation and phosphorylation. **a** Representative images of TUNEL (red) staining in Flag-Olig2 expressing (green) Neuro-2a cells. Neuro-2a cells overexpressing Flag-Olig2 (WT, 3KR, TPN, TPM, 3KR-TPN and 3KR-TPM) were incubated with 20 μM ETO for 36 h, followed by TUNEL staining and immunostaining using anti-Flag antibody. The bar graph shows the percentage of TUNEL^+^ among Flag^+^ cells. Scale bar = 50 μm. TPN triple phospho-null, TPM triple phospho-mimetic. **b** Olig2 SUMOylation has no effect on TSM phosphorylation, but suppresses the overall serine phosphorylation of Olig2. HEK 293T cells were transfected with WT, 3KR Flag-Olig2, His-SUMO1, Myc-Ubc9, or HA-Senp2 as indicated. Lysates were prepared under denaturing conditions and immunoprecipitated with the anti-Flag antibody, followed by western blotting analysis using antibodies against phospho-serine (p-Ser), phospho-S10, S13, S14 (p-TSM) of Olig2, SUMO1, and total Olig2. **c** Olig2 SUMOylation is decreased by S10 phosphorylation. HEK 293T cells were transfected with WT, phospho-defective or phospho-mimetic Myc-Olig2, and Flag-SUMO1 as indicated. Cell lysates were immunoprecipitated with an anti-Myc antibody and then immunoblotted with the anti-SUMO1 and anti-Olig2 antibodies. **d** Olig2 SUMOylation does not affect TSM phosphorylation in vivo. Mouse spinal cord tissue was dissected from *Senp2*^fl/fl^:*Pdgfra*-Cre^ER^ mice administrated with vehicle or tamoxifen (TAM), followed by De-IP and western blotting using anti-Olig2 and anti-p-TSM antibodies, respectively. **e** TSM phosphorylation suppresses Olig2 SUMOylation in vivo. Mouse spinal cord tissue was dissected from WT mice administrated with CHIR-99021 (20 mg/kg, i.p) for 5 days, followed by De-IP and western blotting using antibodies as indicated. The arrowheads indicate SUMOylated Olig2. Images shown are representatives of at least three independent experiments. Bar graphs are presented as means ± s.e.m. of at least three independent experiments. Statistical significance was assessed by Student’s *t* test for two group comparison (**d**, **e**) or one-way ANOVA with Tukey’s multiple comparison test for multiple group comparisons (**a**–**c**). *p < 0.05; **p < 0.01; ***p < 0.001; ns nonsignificant.

The mitogenic and anti-p53 functions of Olig2 are known to be regulated by phosphorylation of TSM [6, 12]. TSM-phosphorylated Olig2 is associated with a transcriptionally active chromatin fraction, where it regulates target gene expression [12]. Interestingly, only overexpression of WT or triple phospho-mimetic Olig2 resulted in protection of Neuro-2a cells from ETO-induced apoptosis. Disrupting either SUMOylation (with 3KR) or TSM phosphorylation (with the triple phospho-null mutant, TPN) was sufficient to completely abolish the protective effect of Olig2 and more importantly, the loss of SUMOylation even diminished the protective effect of TPM (with phosphorylation-SUMOylation double mutant, 3KR-TPM) (Figs. 5a and S3A), indicating that neither SUMOylation nor TSM phosphorylation alone was sufficient to support the antiapoptotic effect of Olig2.

Immunoblotting shows that the phosphorylation of TSM was similar between WT and 3KR (Fig. 5b). For both WT and 3KR Olig2, the TSM phosphorylation levels were unaffected by the co-expression of His-SUMO1, Myc-Ubc9, or HA-Senp2, despite the marked increase in SUMOylation caused by the SUMO conjugating enzyme, Ubc9, and the decrease in SUMOylation resulted from Senp2 (Fig. 5b). However, the overall serine phosphorylation levels of Olig2 were markedly increased in 3KR, as compared with WT Olig2-expressing cells (Figs. 5b and S3B). The phosphorylation nature of these bands was confirmed by using calf intestinal alkaline phosphatase (Fig. S3C). By contrast, the phospho-mimetic S10D and TPM exhibited markedly reduced SUMOylation, while the other forms of phospho-defective and phospho-mimetic mutants were SUMOylated similarly as the WT Olig2 (Fig. 5c). Furthermore, TSM phosphorylation was not altered in spinal cords of *Senp2*^fl/fl^:*Pdgfra*-Cre^ER^ mice, which exhibited upregulated Olig2 SUMOylation upon TAM administration (Figs. 5d and S4). Conversely, reducing the p-TSM level with the GSK3 inhibitor, CHIR-99021 [35], led to an increase in Olig2 SUMOylation (Fig. 5e). Taken together, our data suggest that Olig2 SUMOylation requires S10 dephosphorylation, but the SUMOylation status has no influence on TSM phosphorylation, although it decreases the overall serine phosphorylation of Olig2.

### Olig2 SUMOylation does not alter its stability, subcellular localization, or interaction with other proteins

SUMOylation has been reported to alter subcellular/subnuclear distribution, stability, transcriptional activity, or protein–protein interaction of TFs [36–40]. The protein level of 3KR Olig2 at steady state, as well as the degradation rate, was comparable with that of WT Olig2 (Fig. S5A, B). WT and 3KR mutant displayed similar expression levels and nuclear distribution patterns, no matter treated or not with ETO (Fig. S5C, D) [12].

As a member of the bHLH family, Olig2 not only utilizes its bHLH domain to form homodimers and heterodimers with other bHLH family members, but also interacts physically with some non-bHLH TFs [41–44]. Given that one of the three SUMOylated residues, K112, is located in the bHLH domain, we examined whether SUMOylation changes the interactions of Olig2 with other proteins. Using Myc-Olig2, of which the 3KR mutant showed diminished SUMO1 conjugation (Fig. S5E) just like the Flag-tagged 3KR (Fig. S1E), we showed similar Co-IP of Flag-Olig2 by WT and 3KR Myc-Olig2 (Fig. S5F). The WT and 3KR Myc-Olig2 were also comparable at pulling down a number of Olig2-binding partners, such as Olig1, Nkx2.2, and Sox10 (Fig. S5G–I). Together, these observations indicate that the deficiency in Olig2 SUMOylation has no impact on subcellular distribution or its binding to known Olig2-binding partners.

### Olig2 SUMOylation is required for its gene targeting

**Fig. 6:**
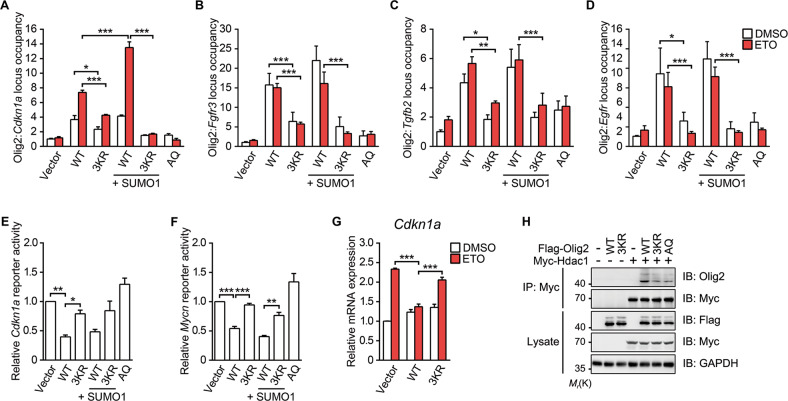
Olig2 SUMOylation is required for its gene targeting. Quantitative ChIP analysis for WT or 3KR Olig2 bound to target genes *Cdkn1a* (**a**), *Fgfr3* (**b**), *Tgfb2* (**c**), and *Egfr* (**d**). Neuro-2a cells transfected with WT Flag-Olig2, 3KR Flag-Olig2, AQ Flag-Olig2, and His-SUMO1 were treated with DMSO or 20 μM ETO for 24 h before being subject to ChIP analysis using anti-Flag antibody. The bar graphs show the ratio of fold enrichment of Olig2 at the target genes over the DMSO-treated vector control. SUMOylation-deficient Olig2 fails to repress luciferase expression driven by the *Cdkn1a* promoter (**e**) or *Mycn* promoter (**f**) in Neuro-2a cells. The luciferase activity for each group was normalized with *Renilla* luciferase activity. **g** Quantitative PCR analysis of *Cdkn1a* mRNA expression. Neuro-2a cells transfected with vector, WT, or 3KR Flag-Olig2 were treated with DMSO or 20 μM ETO for 36 h, followed by RNA extraction and quantitative RT-PCR analysis. The expression level of *Cdkn1a* was normalized to *actin*. **h** 3KR Olig2 is unable to recruit Hdac1. HEK 293T cells transfected with indicated plasmids (WT Flag-Olig2, 3KR Flag-Olig2, AQ Flag-Olig2, and Myc-Hdac1) were subjected to Co-IP analysis using anti-Myc antibody and then immunoblotted with anti-Olig2 antibody. Blot images are representatives of at least three independent experiments. Bar graphs are presented as means ± s.e.m. of at least three independent experiments. Statistical significance was assessed by two-way ANOVA with Tukey’s multiple comparison test for multiple group comparisons. *p < 0.05; **p < 0.01; ***p < 0.001.

The *Cdkn1a* gene, a well-characterized transcriptional target of both Olig2 and p53 [5], encodes a cyclin-dependent kinase 2 inhibitor that mediates p53-dependent cell cycle arrest and thereby determines the fate of tumor cells under genotoxic stress [5, 6, 45, 46]. According to ChIP assays, loss of SUMO modification significantly reduced the occupancy of ectopic Olig2 at the *Cdkn1a* locus (Fig. 6a). Although SUMO1 overexpression increased the occupancy of WT Olig2 at the *Cdkn1a* locus 24 h after ETO stimulation, it failed to affect the binding of 3KR Olig2 to this site (Fig. 6a). These suggest that SUMOylation is required for Olig2 binding to the *Cdkn1a* promoter in response to ETO treatment. As expected, the DNA-binding deficient mutant AQ (K119A/R120Q) [10] did not bind to the *Cdkn1a* locus (Fig. 6a).

Previous studies have also indicated *Fgfr3*, *Tgfb2*, and *Egfr* to be direct genetic targets of Olig2 [12]. Accordingly, we observed the binding of WT Olig2 at the *Fgfr3*, *Tgfb2*, and *Egfr* loci, which was significantly decreased for the 3KR and AQ mutants (Fig. 6b–d). To exclude the possibility that the conformation change associated with the 3KR mutation, rather than Olig2 deSUMOylation, affected its DNA binding, we employed the conjugation-deficient SUMO1 G97A mutant (SUMO1^GA^). As shown in Fig. S6A–D, the DNA-binding abilities of WT Olig2 to all four DNA loci were impaired by co-expression of SUMO1^GA^ to similar extents as the 3KR mutation.

On the other hand, WT Olig2 suppressed the luciferase activities driven by *Cdkn1a* and *Mycn* promoters, respectively, while that of 3KR failed to do the same (Fig. 6e, f), consisting with the marked decrease in *Cdkn1a* transcription (Fig. 6g) and subsequently downregulation of p21 protein (Fig. S6E) in response to ETO in presence of Olig2 WT, but not 3KR. These results provide evidence that Olig2 SUMOylation is required for its transcriptional activity.

Multiple lines of evidence indicate that the nucleosome remodeling and deacetylase (NuRD) complex is recruited to the promoter region by TFs, especially oncogenic TFs, to enhance the transcriptional repression of target genes [47]. As shown in Fig. 6h, both 3KR and AQ Olig2 exhibited marked decreases in the interaction with Hdac1, a core component of NuRD complex [12], as compared with WT Olig2, demonstrating that the SUMOylation deficiency disables Olig2 to effectively recruit Hdac1 to target genes. Collectively, these results strongly support the conclusion that SUMO1 modification of Olig2 is required for transcription suppression.

### Olig2 SUMOylation antagonizes p53 recruitment to *Cdkn1a* promoter

**Fig. 7:**
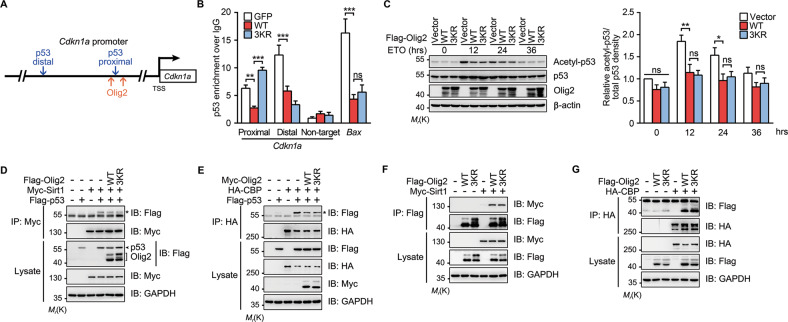
Olig2 SUMOylation antagonizes p53 recruitment to *Cdkn1a* promoter. **a** Schematic showing p53 and Olig2-binding sites in *Cdkn1a* promoter region. Two p53 binding sites and two Olig2-binding sites are indicated with blue and orange arrows, respectively. TSS transcription start site. **b** Quantitative ChIP analysis for p53 occupancy at *Cdkn1a* and *Bax* loci under genotoxic stress. U87-GFP, U87-Olig2^WT^, and U87-Olig2^3KR^ cells were treated with 200 μM TMZ as in Fig. 3b, but for 24 h. Cells were then subjected to ChIP analysis using an anti-p53 antibody. The bar graph shows the ratio of fold enrichment of p53 at the target genes over IgG control. **c** Olig2 represses p53 acetylation in a SUMOylation-independent manner. Neuro-2a cells overexpressing vector, WT, or 3KR Flag-Olig2 were incubated with 20 μM ETO for indicated periods. Cell lysates were subjected to western blotting with antibodies against acetylated p53 (Lys379) and total p53. **d**, **e** Olig2 inhibits the association of p53 with CBP, but not with Sirt1. HEK 293T cells were transfected with WT, 3KR Flag-Olig2 (**d**, or Myc-Olig2 in **e**), Myc-Sirt1, or HA-CBP and subjected to Co-IP analysis using anti-Myc antibody (**d**) or anti-HA antibody (**e**), followed by western blotting using antibodies as indicated. **f**, **g** Olig2 SUMOylation does not affect its interaction with Sirt1 or CBP. HEK 293T cells were transfected with WT or 3KR Flag-Olig2 with Myc-Sirt1 (**f**) or HA-CBP (**g**). Cell lysates were immunoprecipitated with anti-Flag antibody (**f**) or anti-HA antibody (**g**), followed by western blotting using antibodies as indicated. The asterisks indicate co-immunoprecipitated p53. Blot images are representatives of at least three independent experiments. Bar graphs are presented as means ± s.e.m. of at least three independent experiments. Statistical significance was assessed by one-way ANOVA with Tukey’s multiple comparison test for multiple group comparisons. *p < 0.05; **p < 0.01; ***p < 0.001; ns nonsignificant.

Deletion of p53 protects cells from genotoxic damage [6], implicating the promitogenic and antiapoptotic functions of p53. Accordingly, we confirmed the p53-dependent regulation of Olig2 in p53-null (*TP53*^−/−^) HCT-116 cells, in which neither *Cdkn1a* mRNA nor caspase-3 cleavage was induced by ETO, and hence Olig2 overexpression had no further protective effect (Fig. S7A, B). Although no convincing evidence exists for a direct interaction between Olig2 and p53 proteins [6, 12], ChIP-seq studies have shown that Olig2 binds to two promoter/enhancer elements of the *Cdkn1a* promoter region franking the proximal p53-binding site (Fig. 7a) [5]. Thus, we tested the possibility that Olig2 antagonizes the recruitment of p53 to *Cdkn1a* promoter using the ChIP assay. In TMZ-treated U87-MG cells, p53 occupancy at the *Cdkn1a* promoter region encompassing both the proximal p53-binding site and the two franking Olig2-binding sites [5] was dramatically lower in WT Olig2-expressing cells than in cells that expressed the vector control or the 3KR mutant (Fig. 7b). This indicates that Olig2 binding occludes p53 recruitment to this region of the *Cdkn1a* promoter and consistent with the results shown in Fig. 6a.

Surprisingly, both WT and 3KR Olig2 significantly suppressed p53 occupancy at the distal binding site of the *Cdkn1a* promoter when compared with the vector control (Fig. 7b). Given that the expression of WT and 3KR Olig2 also similarly suppressed the binding of p53 to the *Bax* locus (Fig. 7b), a proapoptotic gene target of p53, but not Olig2 [5, 12, 48], this SUMOylation-independent inhibitory effect of Olig2 on p53 recruitment to its gene targets most likely represents a separate mechanism from competitive bindings between p53 and Olig2 to the same promoter region. Indeed, an early study showed that Olig2 inhibits the acetylation of p53 and consequently suppresses the cell response to genotoxic damage [6]. Accordingly, we detected comparable inhibition of ETO-induced p53 acetylation by WT and 3KR Olig2 (including 3KR-TPM) (Figs. 7c and S7C), indicating that SUMOylation is not involved in the regulation of p53 acetylation by Olig2. Acetylation of p53 catalyzed by p300/CBP, as well as deacetylation by Sirt1, are critical for p53-mediated transcriptional activation in DNA-damage response [49, 50]. Co-expression of WT and 3KR Olig2 decreased the physical associations of p53 with CBP, but not with Sirt1 (Fig. 7d, e). At the same time, the associations between Olg2 and Sirt1 and CBP, respectively, were not affected by the loss-of-SUMOylation mutation (Fig. 7f, g). Taken together, our results demonstrate that Olig2 suppresses *Cdkn1a* activation by p53 through at least two separate mechanisms: (1) a SUMOylation-dependent one that involves direct binding of Olig2 to the *Cdkn1a* promoter to occlude p53 binding to the proximal site and (2) a SUMOylation-independent one that inhibits p53 acetylation through disrupting p53–CBP interaction (Fig. S7D).

## Discussion

Olig2, a CNS-specific TF, is well known to support tumor development. TSM-phosphorylated Olig2 enhances chemoresistance and radioresistance of human gliomas, but the precise mechanisms remain elusive [6, 11, 12]. Numerous studies have demonstrated that PTMs, such as phosphorylation, acetylation, and SUMOylation, serve as a vital link in DNA-damage response [27, 51, 52]. Here we demonstrate Olig2 as a SUMOylation target that undergoes covalent SUMO1 conjugation. Both SUMOylation and TSM-phosphorylation are required for the function of Olig2 to prevent the genotoxic-induced apoptosis. The SUMOylation of Olig2 supports its DNA-binding ability, which in turn inhibits p53-mediated gene targeting upon genotoxic damage.

### Crosstalk between Olig2 SUMOylation and its phosphorylation

PTMs have emerged as important regulatory mechanisms in cellular responses to various stimuli [52, 53]. SUMOylation represents one of the most dynamic PTMs, with a diverse repertoire of effects ranging from protein localization, interaction, to transcriptional regulation [54]. In mice, TSM phosphorylation of Olig2 is essential for tumor growth and resistance to genotoxic damage [6, 11]. Here, we show that both SUMOylation and TSM phosphorylation are important to the antiapoptotic function of Olig2 (Figs. 5a and S3A) [6]. TPM, despite the decreased SUMOylation, still exhibited the antiapoptotic function when placed in the WT (but not the 3KR) background (Figs. 5a and S3A). While the antiapoptotic effect of WT-TPM may be explained by the “SUMO Enigma,” which claims that only minimal SUMOylation of a particular protein is necessary to achieve the maximal functional effects [55], the failure of WT-TPN to exert such an effect may result from the fact that Olig2 with unphosphorylated TSM binds to a transcriptionally inactive chromatin domain, making it inaccessible to target genes [12]. Thus, despite the apparent hyper-SUMOylation (Fig. 5c), WT-TPN is not expected to interact with its genetic target to elicit function.

### Olig2 SUMOylation in transcription repression

SUMO modification is more often associated with transcription repression [27, 56]. Although the SUMOylation level of a particular TF (e.g., Olig2) at steady state is extremely low and hardly detectable (Fig. 1c, d), the repression effect of SUMO appears to be maximal (Fig. 6a–f) [55, 57]. A general model has been developed to accommodate these observations: SUMOylated TFs are assembled into a repression complex (e.g., the NuRD complex) in a SUMO-dependent manner. Once the complex is formed, TFs are deSUMOylated by SENPs but retained in the complex. In this case, SUMO modification initiates transcription repression, but is not required to maintain the repression [55, 57]. The recruitment of transcription repressors to SUMOylated TFs is reported to be facilitated by the SUMO interacting motifs (SIMs) located in the repressors [28, 58, 59]. In fact, a bioinformatic predictor for SUMOylation sites and SIMs, JASSA [30], identified four potential SIMs in Olig2 interacting transcription repressors Hdac1 and Mta2 [12], which are highly conserved among species.

Given that one of the SUMO-acceptor sites K112 is located in the basic region of bHLH domain [60], SUMOylation might facilitate DNA-Olig2 interaction by being directly involved in DNA binding [61], or serving as a bridge linking basal transcriptional machinery, TFs, or coregulators and thereby recruiting Olig2 to DNA [55, 62]. We show that 3KR Olig2 exhibits decreased binding capacity to Hdac1, similar to the AQ mutant (Fig. 6h), but the binding to the transcription activator, CBP, is unaffected (Fig. 7g). Thus, we propose that SUMO1 covalently conjugated Olig2 interacts with DNA to form an Olig2:DNA complex that acts as a scaffold to recruit the transcription repression complex (e.g., the NuRD complex) to DNA. Interestingly, the SUMOylated Olig2 may be rapidly deconjugated by Senps, resulting in the abundant presence of non-SUMOylated Olig2 in the complex, which retains the transcription repression. Further studies are warranted to unravel the precise mechanisms on how SUMOylated Olig2 initiates transcription suppression.

### Olig2 SUMOylation and p53 activation

Olig2 is expressed in multiple grades of human glioma [63–65] and is required for gliomagenesis in murine models of glioma [8]. It has been reported that Olig2 suppresses p53 gene targeting in irradiation-induced apoptosis by inhibiting acetylation of p53 [6]. Interestingly, the protective functions of Olig2 SUMOylation require E-box binding, as the DNA-binding deficient 3KR fails to protect cells from DNA damage (Figs. 2 and 6) [12], and repression of p53 acetylation alone is not sufficient to inhibit DNA-damage response (Figs. 2 and 7c). ChIP-seq studies have revealed a pair of E-box elements near one of the canonical p53-binding site on the *Cdkn1a* promoter [5]. Accordingly, the proximity ligation assay has indicated that Olig2 stays very close to p53 in the open chromatin, but the Olig2–p53 complex has not been successfully identified [6, 12]. Our findings provide more detailed insights on p53 inhibition by Olig2 wherein SUMOylation promotes Olig2:*Cdkn1a* interaction that occludes p53 binding to the *Cdkn1a* promoter (Figs. 6a and 7b). In addition, Olig2 (either SUMOylated or non-SUMOylated) disrupts p53 acetylation by inhibiting the CBP–p53 interaction (Fig. 7c, e), which suppresses transactivation of other p53 target genes (e.g., *Bax*). Our results indicate the importance of Olig2-mediated transcription repression in DNA-damage response, which might even overwhelm its p53-acetylation suppressing function. However, a genome-wide screen for Olig2 target genes in Olig2-null neural stem cells has identified only four Olig2-repressible genes associated with p53 signaling axis, three of which are poorly characterized [5]. Numerous DNA-damage responsible genes are upregulated or downregulated [46], wherein almost no genetic target of Olig2 has been well studied.

In summary, we demonstrate that Olig2 is SUMOylated and the SUMOylation is essential for Olig2 suppression of p53-dependent DNA-damage response. The protective effect of Olig2 SUMOylation against DNA-damage response is mediated by antagonizing p53 recruitment to the *Cdkn1a* promoter, which consequently prevents genotoxic drug-induced cell cycle arrest and apoptosis. Our work uncovers a SUMOylation-dependent regulatory mechanism of Olig2 in DNA-damage response. The more in-depth understanding of the Olig2 SUMOylation regulated pathway described here at the molecular level will facilitate the design of new strategies for treatment of tumor resistant to genotoxic stress, such as GBM.

## Supplementary information

Figure S1

Figure S2

Figure S3

Figure S4

Figure S5

Figure S6

Figure S7

Supplementary Figure Legends
