# Supplementary material for: Olig2 SUMOylation protects against genotoxic damage response by antagonizing p53 gene targeting
Source: Cell Death Differ. 2020 Jun 1;27(11):3146–61. doi: 10.1038/s41418-020-0569-1 (PMC7560653; doi:10.1038/s41418-020-0569-1)
Supplement: Supplementary file 8 — Supplementary Figure Legends [file 41418_2020_569_MOESM8_ESM.docx]

**Supplementary Figure Legends**

**Fig. S1. Determination of Olig2 SUMOylation.** (**A**) Olig2 is modified by SUMO1 *in vivo*. Mouse spinal cord tissues were homogenized and lysed under denaturing conditions, followed by immunoprecipitation with mouse SUMO1 affinity gel, with mouse normal IgG as the negative control. Western blotting was performed on the eluted products using rabbit anti-Olig2 and rabbit anti-SUMO1 antibodies. (**B**) Comparison of the molecular weight between ectopic Olig2 and endogenous Olig2. U87, whole cell lysate of U87-MG cells; TG, lysate of mouse trigeminal ganglion; Flag-Olig2, whole cell lysate of HEK 293T cells transiently transfected with Flag-Olig2. The lysate was diluted ~300 times with 1 × SDS loading buffer before subjecting to SDS-PAGE; SC, lysate of mouse spinal cord; SC+Flag-Olig2, whole cell lysate of HEK 293T cells transiently transfected with Flag-Olig2, diluted ~300 times with spinal cord lysate. U87 and TG were used as negative controls. The arrowheads and asterisk indicate ectopic Olig2 and endogenous Olig2, respectively. (**C**) Predicted SUMO modification sites in mouse Olig2 by GPS-SUMO, SUMOplot and JASSA. (**D**) Alignment of mammalian Olig2 orthologs in areas flanking K27, K76, and K112. (**E**) Conjugation of Olig2 by endogenous SUMO1. HEK 293T cells were transfected with control vector, WT and 3KR Flag-Olig2 without His-SUMO1. Cell lysates were subject to De-IP using the Flag antibody and followed by immunoblotting with anti-SUMO1 and anti-Olig2 antibodies. The arrowhead indicates SUMOylated Olig2. Blot images are representatives of at least three independent experiments.

**Fig. S2. Mapping Olig2 SUMOylation sites by LC-MS/MS analysis.** Flag-Olig2 proteins were purified from HEK 293T cells that co-expressed Flag-Olig2 and His-SUMO1-T95R by immunoprecipitation with the anti-Flag antibody. The products were digested with trypsin protease and subjected to LC-MS/MS analysis. (**A**) MS/MS spectrum of tryptic peptide containing K27 subject to collision-activated dissociation (CAD). The arrow indicates the 114 Da increase on the Serine-Lysine (SK) dipeptide produced by CAD from the N-terminus due to covalent attachment of the diglycine (GG) from His-SUMO1-T95R, suggesting that K27 is a SUMOylation site. (**B**) MS/MS spectrum of tryptic peptide containing K76 subject to CAD. The arrow indicates the 114 Da increase on the Lysine (K) produced by CAD from the C-terminus due to covalent attachment of GG, suggesting that K76 is a SUMOylation site.

**Fig. S3. Both SUMOylation and TSM phosphorylation of Olig2 are required for its anti-apoptotic function.** (**A**) Representative images of TUNEL (red) staining in Flag-Olig2 expressing (green) Neuro-2a cells. Neuro-2a cells overexpressing WT, 3KR, TPN, TPM, 3KR-TPN, 3KR-TPM Flag-Olig2 and His-SUMO1 were incubated with 20 μM ETO for 36 hrs, followed by TUNEL staining and immunostaining using anti-Flag antibody. The bar graph shows the percentage of TUNEL^+^ among Flag^+^ cells. Scale bar = 50 μm. (**B**) Olig2 SUMOylation inhibits its overall phosphorylation of serine residues. Summary data for the experiment shown in Fig. 5B of p-Ser immunoblots. (**C**) CIAP treatment validates the specificity of anti-p-Ser and anti-p-TSM antibodies. IP products bound to the beads were treated with vehicle or CIAP at 37°C for 1 hr before being subjected to Western blotting (diagram). Bar graphs are presented as means ± s.e.m. from three independent experiments. Statistical significance was assessed by one-way ANOVA with Tukey’s multiple comparison test for multiple group comparisons. *p＜0.05; **p＜0.01; ***p＜0.001; ns, non-significant.

**Fig. S4. Generation of *Senp2*^fl/fl^:*Pdgfra*-Cre^ER^ mice.** (**A**) Diagram depicting generation of *Pdgfra*-Cre-mediated excision of *Senp2* exon 13 and 14 in oligodendrocyte progenitor cells (OPCs). (**B**) Verification of *Senp2* (red) deletion in Olig2^+^ (green) OPCs by immunostaining. Spinal cord was dissected from vehicle (corn oil) or TAM treated mice and immunostained with antibodies against Senp2 and Olig2. DAPI was used to stain cell nuclei. Arrowheads indicate the nuclei with Olig2 expression in presence (upper) and absence (lower) of Senp2. Scale bar = 20 μm.

**Fig. S5. Olig2 SUMOylation does not alter its stability, subcellular localization or ability to interact with its protein partners.** (**A**) Olig2 SUMOylation does not alter its expression level at steady state. HEK 293T cells were transfected with indicated plasmids and then lysed for Western blotting analysis. (**B**) Olig2 SUMOylation does not affect its degradation rate. HEK 293T cells were transfected with WT or 3KR Flag-Olig2 for 24 hrs and then incubated with 20 μg/ml CHX for the indicated time periods, before being analyzed by Western blotting. Data points are means ± s.e.m. of three independent experiments. (**C**) Co-labeling of Flag-Olig2 (green) and nuclei (DAPI, blue) in Neuro-2a cells. Cells transfected with WT or 3KR Flag-Olig2 were treated with DMSO or 20 μM ETO for 36 hrs for immunostaining. Scale bar = 20 μm. (**D**) Subcellular distribution of WT and 3KR Olig2. Cell components were separated into cytoplasmic and nuclear fractions and subjected to Western blotting analysis with anti-Olig2 antibody. Histone H3 and GAPDH were used as loading controls for nuclear and cytoplasmic fractions, respectively. (**E**) Identification of endogenous SUMO1-conjugated Myc-Olig2. WT or 3KR Myc-Olig2 were transfected into HEK 293T cells. Cell lysates were immunoprecipitated using anti-Myc antibody and immunoblotted with anti-Olig2 and anti-SUMO1 antibodies. Arrowhead indicates SUMOylated Olig2. (**F**-**I**) Protein binding properties of Olig2. HEK 293T cells were transfected with WT or 3KR Myc-Olig2, together with Flag-Olig2 (WT or 3KR) (**F**), HA-Olig1 (**G**), HA-Nkx2.2 (**H**) or HA-Sox10 (**I**). Cell lysates were immunoprecipitated using anti-Myc antibody and immunoblotted with anti-Flag, anti-Olig2, and anti-HA antibody, as indicated. Images are representatives of at least three independent experiments.

**Fig. S6. Deficiency of Olig2 SUMOylation impairs its DNA binding ability.** (**A**-**D**) Quantitative ChIP analysis for WT or 3KR Olig2 bound to target genes *Cdkn1a* **(A)**, *Fgfr3* **(B)**, *Tgfb2* **(C)** and *Egfr* **(D)**. Neuro-2a cells transfected with WT Flag-Olig2, 3KR Flag-Olig2, His-SUMO1^WT^ or His-SUMO1^GA^ were treated with DMSO or 20 μM ETO for 24 hrs before being subjected to ChIP analysis. The bar graphs show the ratio of fold enrichment of Olig2 at the target genes over the DMSO-treated vector control. (**E**) p21 is downregulated in the presence of SUMOylated Olig2 under DNA damage. Neuro-2a cells transfected with vector, WT or 3KR Flag-Olig2 were treated with DMSO or 20 μM ETO for 36 hrs, followed by Western blotting. Blot images are representatives of at least three independent experiments. Bar graphs are presented as means ± s.e.m. for at least three independent experiments. Statistical significance was assessed by two-way ANOVA with Tukey’s multiple comparison test for multiple group comparisons. *p＜0.05; **p＜0.01; ***p＜0.001.

**Fig. S7. The anti-apoptotic function of Olig2 requires p53.** (**A**, **B**) p53-null (*TP53*^-/-^) and control (*TP53*^+/+^) HCT-116 cells overexpressing vector, WT or 3KR Myc-Olig2 were treated with DMSO or 20 μM ETO for 36 hrs and subjected to quantitative PCR (**A**) and Western blotting analysis (**B**). (**C**) Olig2 inhibits p53 acetylation, but not total p53 expression, in a SUMO-independent manner. Neuro-2a cells transfected with vector, WT Flag-Olig2 and the indicated mutants were treated with 20 μM ETO for 36 hrs, followed by Western blotting for acetylated and total p53. (**D**) Schematic model of Olig2 SUMOylation in the regulation of DNA damage response. The protective effect of Olig2 SUMOylation against DNA damage response is mediated by at least two separate mechanisms: 1) a SUMOylation-dependent one that involves direct binding of Olig2 to the *Cdkn1a* promoter to occlude p53 binding to the proximal site and 2) a SUMOylation-independent one that inhibits p53 acetylation through disrupting p53-CBP interaction. Blot images are representatives of at least three independent experiments. Bar graphs are presented as means ± s.e.m. for three independent experiments. Statistical significance was assessed by two-way ANOVA with Tukey’s multiple comparison test for multiple group comparisons. ***p＜0.001.
